# Psychometric Properties of the Persian Version of the Tinnitus Handicap Inventory (THI-P)

**Published:** 2015-03

**Authors:** Mir Mohammad Jalali, Robabeh Soleimani, Mahnaz Fallahi, Mohammad Aghajanpour, Masoumeh Elahi

**Affiliations:** 1 *Department of Otorhinolaryngology, Amiralmomenin Hospital, Guilan University of Medical Sciences, Guilan, Iran.*; 2 *Department of Psychiatry, Shafa Hospital, Guilan University of Medical Sciences, Guilan, Iran.*

**Keywords:** Anxiety, Depression, Questionnaires, Reproducibility of results, Tinnitus, Psychometrics

## Abstract

**Introduction:**

Tinnitus can have a significant effect on an individual’s quality of life, and is very difficult quantify. One of the most popular questionnaires used in this area is the Tinnitus Handicap Inventory (THI). The aim of this study was to determine the reliability and validity of a Persian translation of the Tinnitus Handicap Inventory (THI-P).

**Materials and Methods:**

This prospective clinical study was performed in the Otolaryngology Department of Guilan University of Medical Sciences, Iran. A total of 102 patients aged 23–80 years with tinnitus completed the (THI-P). The patients were instructed to complete the Beck Depression Inventory (BDI) and the State-Trait Anxiety Inventory (STAI). Audiometry was performed. Eight-five patients were asked to complete the THI-P for a second time 7–10 days after the initial interview. We assessed test–retest reliability and internal reliability of the THI-P. Validity was assessed by analyzing the THI-P of patients according to their age, tinnitus duration and psychological distress (BDI and STAI). A factor analysis was computed to verify if three subscales (functional, emotional, and catastrophic) represented three distinct variables.

**Results:**

Test–retest correlation coefficient scores were highly significant. The THI-P and its subscales showed good internal consistency reliability (α = 0.80 to 0.96). High-to-moderate correlations were observed between THI-P and psychological distress and tinnitus symptom ratings. A confirmatory factor analysis failed to validate the three subscales of THI, and high inter-correlations found between the subscales question whether they represent three distinct factors.

**Conclusion:**

The results suggest that the THI-P is a reliable and valid tool which can be used in a clinical setting to quantify the impact of tinnitus on the quality of life of Iranian patients.

## Introduction

Tinnitus is a phantom auditory perception or sensation (1,2), with a prevalence in adults of 10–15% (3,4). The mechanism and pathophysiology of tinnitus is not well defined. Tinnitus can cause emotional distress in patients and affect their quality of life. Although some people with tinnitus experience depression, irritation, distraction or insomnia (5), tinnitus is not a significant problem in 80% of cases (6). As tinnitus is largely a subjective complaint, it is important to identify patients whose life is affected by the condition. One of the most significant efforts to understand the complexity of problems in tinnitus patients was undertaken by Newman et al. who introduced the Tinnitus Handicap Inventory (THI) questionnaire (7). 

This questionnaire consists of 25 items and three subscales: the performance subscale (12 items), the emotional subscale (8 items), and the catastrophic subscale (5 items). These scales represent role and physical functioning, psychological distress, and depression and loss of control, respectively. Each item has three potential answers: 4 points for ‘yes’, 2 points for ‘sometimes’, and 0 points for ‘no’. The total score for the test ranges between 0 (no tinnitus handicap) and100 (the worst possible annoyance). In the full version of the THI (0–100 points), scores ranging between 0 and 16 points are associated with no handicap, and scores of 18 points or more reflect some degree of handicap (i.e., mild, moderate, severe, or catastrophic categories) (8). Studies have shown that THI is a robust and appropriate psychometric tool to measure the impact of tinnitus on daily life (8) and to determine the outcomes of various treatments (9-11).

THI has high internal consistency, reliability, convergent validity, and construct reliability (12). This questionnaire has been translated into various languages, including Turkish, Danish (THI-DK), Spanish, Korean, Portuguese, Italian, Singaporean languages, Chinese(Mandarin: THI-CM; Cantonese: THI-CH), Japanese, and Filipino (12-22). It has demonstrated adequate reliability and validity in all of these translations. In 2011, Mahmoudian et al. translated this questionnaire into Persian and applied it in 112 patients with tinnitus. The researchers did not find any significant differences in gender, age, hearing impairment, and total score and subscales of Persian version of THI (THI-P). Pearson product-moment correlations revealed adequate test–retest reliability for the THI-P (r=0.96). Cronbach’s α coefficient indicated adequate internal stability of the THI-P (r=0.943), with a total–item correction varying between r=0.939 and r=0.944, indicating its reproducibility (23).

To the best of our knowledge, there is no study evaluating the construct validity of the THI, nor any report of its psychometric properties. The aim of this study was to continue the work of Mahmoudian and colleagues concerning the reliability and validity of the Persian version of the questionnaire, and to undertake the following: 1) assess the reliability and validity of the Persian version of THI for measuring the consequences of tinnitus; 2) investigate the complex relationship between tinnitus and psychological distress in adult outpatients suffering from tinnitus; 3) assess the basic psychometric properties of the THI-P.

## Materials and Methods

This study is a psychometric validation of the THI by Newman, conducted from March to September 2013. 

The original version of the THI (THI-US) was translated into Persian (THI-P) using a translation-back translation method (23). Two native speakers of Persian, bilingual in English and Persian, separately performed the forward translation independently of one another. Two of the investigators (JMM, SR) performed a synthesis of the two translations. A fluent English-speaking person who was ignorant of the original version then back translated this version into English. An expert team composed of two of the investigators (JMM, SR) and a bilingually experienced translator reviewed the preliminary version of the THI. The wording of individual items was then adjusted based on a comparison of the original and the back-translated version. Three audiologists further verified the appropriateness of the translation. A form with Persian and English versions of all the 25 items was given to them. Each item was to be rated as either ‘appropriate’ or ‘inappropriate’. If an item was deemed inappropriate, the judges would have to suggest corrections. If two out of three judges found an item in the THI-P appropriate, the translation was considered correct. Minor alterations were carried out according to the answers obtained from the three audiologists, and our Persian version of the THI was then finalized.

*Subjects*

The final version of the THI-P was administered to 102 tinnitus patients (62 males, 40 females, aged 20–80 years) recruited consecutively at the Department of Otorhinolaryngology, Amiralmomenin Hospital, Guilan University of Medical Sciences (GUMS) after receiving otoneurological and audiological evaluations. Inclusion criteria were sensorineural-related tinnitus, and we excluded cases of concurrent external and middle ear diseases and temporoman- dibular joint (TMJ) disorders. Patients with dizziness, vertigo, mental retardation or psychiatric disorders, head and neck disease, or any organic disease (for example vascular disease) were excluded. We considered the educational level and hand preference of patients. The study was approved by the ethics committee of GUMS and was conducted in accordance with Helsinki Declarations. Informed consent was obtained from all subjects before testing. Test–retest reliability was assessed following a second investigation. We asked the patients to complete the inventory again 7–10 days after the first investigation. Eighty-five of 102 patients (49 males, 36 females, aged 23–80 years) completed the second inventory. None of the included patients were given any intervention.

Tonal and vocal audiometry and impedance tests were carried out in all patients. Tinnitus pitch and loudness matches were determined using the two-alternative forced-choice method. A pair of tones was presented and the subject was asked to identify which tone best matched the pitch of their tinnitus. Loudness matches were tested using the bracketing approach with 1-dB increments. The tones were presented in the ear contralateral to the predominant or louder tinnitus (24). If the tinnitus was equally loud on both sides, the ear with the better hearing was used as the test ear. If there was no difference in hearing acuity, the test ear was chosen randomly.

*Assessment of Tinnitus Severity*

Tinnitus severity was assessed by asking patients to complete a visual analogue scale (VAS) measuring the perceived loudness of their tinnitus (tinnitus loudness). The VAS scales consisted of a 10-cm line with endpoints anchored as total absence and maximum tinnitus loudness. 

*Additional Measures*

As mentioned earlier, the THI consists three subscales: functional, emotional and catastrophic which evaluate physical functioning, affective responses to tinnitus, and ability to cope and fear of a grave disease. Then we considered following questionnaires for evaluating construct validity. The 21-item self-report Beck Depression Inventory (BDI) was used to assess current depressive symptoms (25). Its validity and reliability have been demonstrated in Iran previously (Cronbach’s α: 0.89, test–retest correlation: 0.94) (26). Each item has four statements describing increasing levels of severity, and the total score ranges from 0 to 63. Scores of 10 or more indicate the presence of depressive symptoms (27). 

Anxiety was assessed using the Persian version of the Spielberger State-Trait Anxiety Inventory (STAI). The STAI scale consists of 40 statements describing various emotional states. Twenty of these statements require the subjects to describe their emotional reaction in terms of anxiety at a particular moment or period in time (state anxiety). Statements are scored on a 4-point scale of increasing intensity, from ‘not at all’ to ‘very much so’ (with scores of 0–3, respectively). The other 20 items require the subject to describe how they generally feel and their general response to situations perceived as threatening (trait anxiety). These items are also scored on a 4-point intensity scale, from ‘almost never’ to ‘almost always’. For both sections, possible cumulative scores for each scale range from 0 (not anxious) to 60 (high anxiety). Scores of 40 or more indicate the presence of anxiety symptoms (28). The Persian version of STAI has been used in previous studies and its validity and reliability have been demonstrated. The value of Cronbach’s α coefficient for state/trait anxiety were 0.70 and 0.78, respectively (29, 30).

*Statistical Analysis*

For this trial, we evaluated the suitability of the sample using the Bartlett test of Sphericity. The value for this test was less than 0.001 (chi-square = 1870.23, df = 300), indicating that there are correlations in the dataset that are appropriate for factor analysis. Also, we assessed sampling adequacy using the Kaiser-Meyer-Olkin (KMO) measurement. KMO varies from 0 to 1.0 and the overall KMO value should be 0.60 or higher to proceed with factor analysis; with a value greater than 0.90 considered outstanding. In this study, the KMO value was 0.93.

For external reliability of the questionnaire, we used test–retest reliability. For analysis of internal reliability of the questionnaire, Cronbach’s α coefficient was calculated. Principal component analysis with varimax rotation was used to validate the THI factor structure. Items were retained if they had loadings equal to or greater than ±0.40 and an eigenvalue ≥ 1. Factor loading is the most significant factor in the interpretation of the principal component analysis and a value of ±0.4 is considered highly significant. Also a criterion of eigenvalues is to determine the number of factors.

An independent t-test was computed to test the hypothesis that the THI score and its subscales could differ between males and females. Pearson’s product moments were calculated between the total score of the THI-P and the scores of its subscales, age and pure tone average (PTA) to assess correlations.

Pearson’s product moments were also calculated between the total score of the THI-P and the scores of its subscales, as well as between the scores of the Beck, the STAI, and VAS to test convergent validity. 

Statistical significance was set at P<0.05 in all analyses, for which the statistical software SPSS 21.0 version was used. We performed a confirmatory factor analysis using AMOS. In confirmatory factor analysis (CFA), we specified a model, indicating which variables loaded on which factors and which factors were correlated.

## Results

*Participant Characteristics*

The mean age of the participants was 50.25 ± 13.40 years (range, 20–80 years) and 60.8% of cases were male. All patients suffered from continuous uni- or bilateral tinnitus with a mean duration of 2.8 years (range 6 months to 18 years, median 1.8 years).

The mean PTA hearing threshold, calculated over 0.5, 1, 2, and 4 kHz across both ears, ranged from 1.25–76.25 dB HL (mean=20.36 dB). In 50 cases (49%) the audiograms revealed bilateral sloping high-frequency hearing losses. Thirty-seven patients (36.3%) had bilateral normal hearing (PTA ≤ 25 dB HL) and 15 (14.7%) showed a unilateral sensorineural hearing loss. In terms of PTA hearing threshold, a low non-significant correlation was found between the mean hearing threshold and tinnitus handicap scores. Also, there was a low correlation between tinnitus duration and THI-P score (P<0.05). We found significant medium-to-high correlations between VAS, BDI, STAI and THI-P scores (all correlation were significant at the 0.01 level).

The majority of subjects (47%) were 46–65 years old, followed by those aged 26–45 years (34%) the 65 years and older (15%). The lowest concentration of subjects was found in the 20–25-year age bracket (4%). As was the case for the original version of the THI, no correlations between age and tinnitus handicap score were observed in the THI-P (ρ = 0.138, P= 0.17).

*Tinnitus Characteristics*

Most of the patients suffered from unilateral tinnitus (60%), while 40% suffered from bilateral tinnitus. Tinnitus pitch was most frequently matched at 4200 Hz, while mean loudness matching was at the 8-dB sensation level (SL) (standard deviation [SD]=4.6). All loudness matches across all handicap grades were at the 0–12 dB SL. A significant correlation was found between the duration of the tinnitus and tinnitus handicap scores (P=0.228, P=0.02). The majority of cases were classified as slight (28.52%), followed by mild and moderate (22.54% each), severe and catastrophic (13.72% each). In males, the majority of cases were concentrated in the mild (25.8%) and moderate (25.8%) grades while in females most of the cases were slight (39.9%). Mean total THI in male and female participants was 42.16 and 37.95, respectively. Gender differences in perceived tinnitus handicap were examined using t-tests for independent samples. As in the case for the original version of the THI, no significant effect of gender was observed in the THI-P (P>0.05).

*Reliability*

Table.1 summarizes the endorsement rates for each of the 25 items for the Persian translation (THI-P) and the original version (THI-US). As seen, the endorsement rates for a “Yes”, “Sometimes”, and “No” response ranged from 5.9–47.1%, 11.8–58.8%, and 20.6–75.5%, respectively. The score ranges of the THI-US version were 8–63%,11–49%, and 19–64%, respectively.

The external reliability of the THI-P was examined from its test–retest reliability. The Pearson correlation between the first and second scores on the three subscales (functional, emotional, and catastrophic) and between the first and second total score is presented in (Table. 2). The Pearson correlations ranged between 0.78 (items 4, 23) and 0.99 (item 2). The Pearson correlation was greater than 0.94 for all subscales. The Pearson correlation for the total score was 0.98. These high Pearson correlation values indicate good test–retest reliability.

The internal validity of the THI-P was assessed using Cronbach’s α coefficients. Cronbach’s α coefficient for the total score was 0.96. The item–total correlation ranged between 0.42 (items 2) and 0.79 (item 16). The Cronbach’s α coefficients for the functional, emotional, and catastrophic subscales were 0.91, 0.91, and 0.80, respectively (Table. 3). As can be seen in Table 3, the ranges of item–total correlations for the THI-total and subscales of the THI-P were also comparable with those of the THI-US. As seen in (Table. 4), the mean scores of the Persian translation were generally higher than those of the original version for the THI-total scale and THI-functional subscales. The score ranges of the Persian translation, however, were comparable with those of the THI-US.As seen in (Table.5), the correlations among the total- and subscales of the THI-P were comparable with the correlations found among the scores of the subscales of the original (THI-US) version.

**Table 1:** Endorsement rates (percentage of respondents selecting response) and item–total correlations* of the Persian translation (THI-P) and the original version of the Tinnitus Handicap Inventory (THI-US)**

Item	Endorsement rates (%)			Item-total correlation
	Yes	Sometimes	No	
**The purpose of the scale is to identify the problems your tinnitus may be causing you. Check “Yes”, “Sometimes”, or “no” for each question. Do not skip a question.**				
**1F Because of your tinnitus, is it difficult for you to concentrate?**	44.1 (24)	29.4 (49)	26.5 (27)	0.53 (0.70)
**2F Does the loudness of your tinnitus make it difficult to hear people?**	46.1 (35)	22.5 (35)	31.4 (30)	0.80 (0.22)
**3E Does your tinnitus make you angry? **	37.3 (20)	42.2 (38)	20.6 (42)	0.71 (0.54)
**4F Does your tinnitus make you feel confused? **	6.9 (18)	52.9 (25)	40.2 (57)	0.68 (0.64)
**5C Because of your tinnitus, do you feel desperate? **	11.8 (17)	26.5 (25)	61.8 (58)	0.81 (0.54)
**6E Do you complain a great deal about your tinnitus? **	30.4 (17)	41.2 (26)	28.4 (57)	0.80 (0.63)
**7F Because of your tinnitus, do you have trouble falling asleep at night?**	47.1 (24)	30.4 (38)	22.5 (38)	0.98 (0.48)
**8C Do you feel as though you cannot escape your tinnitus? **	23.5 (60)	46.1 (20)	30.4 (20)	0.87 (0.55)
**9F Does your tinnitus interfere with your ability to enjoy social activities (dinner, movies)?**	14.9 (8)	57.4 (29)	27.7 (63)	0.89 (0.61)
**10E Because of your tinnitus, do you feel frustrated? **	12.7 (29)	37.3 (37)	50 (34)	0.89 (0.77)
**11C Because of your tinnitus, do you feel that you have a terrible disease?**	11.8 (14)	29.4 (23)	58.8 (63)	0.88 (0.48)
**12F Does your tinnitus make it difficult for you to enjoy life? **	11.8(12)	54.9 (26)	33.3 (62)	0.63 (0.69)
**13F Does your tinnitus interfere with your job or household responsibilities?**	13.7 (10)	58.8 (32)	27.5 (58)	0.89 (0.56)
**14F Because of your tinnitus, do you find that you are often irritable?**	35.3 (22)	25.5 (32)	39.2 (46)	0.88 (0.69)
**15F Because of your tinnitus, is it difficult for you to read? **	24.5 (20)	37.3 (29)	38.2 (51)	0.88 (0.48)
**16E Does your tinnitus make you upset? **	36.3 (25)	23.5 (38)	40.2 (37)	0.98 (0.76)
**17E Do you feel that your tinnitus has placed stress on your relationship?**	9.8 (26)	58.8 (20)	31.4 (54)	0.87 (0.53)
**18F Do you feel it difficult to focus your attention away from your tinnitus and on other things?**	22.5 (15)	43.1 (42)	34.3 (43)	0.67 (0.69)
**19C Do you feel that you have no control over your tinnitus? **	26.5 (63)	28.4 (18)	45.1 (19)	0.67 (0.48)
**20F Because of your tinnitus, do you feel tired? **	31.4 (18)	38.2 (23)	30.4 (59)	0.98 (0.58)
**21E Because of your tinnitus, do you feel depressed? **	18.6 (18)	13.7 (26)	67.6 (56)	0.98 (0.63)
**22E Does your tinnitus make you feel anxious? **	12.7 (25)	32.4 (28)	54.9 (49)	0.98 (0.54)
**23C Do you feel that you can no longer cope with your tinnitus?**	12.7 (11)	32.4 (40)	54.9 (49)	0.89 (0.59)
**24F Does your tinnitus get worse when you are under stress? **	23.5 (43)	11.8 (25)	64.7 (32)	0.98 (0.49)
**25E Does your tinnitus make you feel insecure? **	5.9 (16)	18.6 (20)	75.5 (64)	0.88 (0.47)

* F represents items included in the functional subscale, E, items included in the Emotional subscale, and C, items included in the catastrophic response subscale.

** Endorsement rates and item-total correlations of the original (THI-US) version are listed in parentheses.

**Table 2:** Pearson product-moment correlation coefficients, standard error (SE) of measurement, 95% confidence intervals (CIs), mean and standard deviation (SD), range values associated with the test–retest administrations of the Persian version of Tinnitus Handicap Inventory (THI-P) subscales and total scores (N=85).

THI-P	Test	Retest	Correlation Coefficients	S_e_	95% CI	p-value
**Functional (Maximum score: 48)**						
**Mean**	37.9	38.1	0.98	3.0	6.2	
**SD**	26.8	26.8				0.001
**Range**	0-48	0-48				
**Emotional (Maximum score: 32)**						
**Mean**	22.0	21.6	0.98	1.5	2.1	
**SD**	13.5	13.5				0.001
**Range**	0-30	0-28				
**Catastrophic response (Maximum score: 20)**						
**Mean**	10.8	11.0	0.94	1.0	2.0	
**SD**	9.1	9.1				0.001
**Range**	0-18	0-20				
**Total (Maximum score: 100)**						
**Mean**	6.1	6.3	0.98	0.6	1.3	
**SD**	5.4	5.5				0.001
**Range**	4-92	2-94				
						

**Table 3:** Reliability coefficients (Cronbach’s α coefficient) of the Persian translation of the THI (THI-P) and the original version (THI-US).

	THI-P (α)	THI-US (α)	THI-P (Item-total)	THI-US (Item-total)
**THI-total (25 items) **	0.96	0.93	0.42–0.79	0.22–0.77
**Functional (12 items) **	0.91	0.86	0.51–0.75	0.27–0.76
**Emotional (8 items) **	0.91	0.87	0.62–0.79	0.56–0.82
**Catastrophic (5 items) **	0.80	0.68	0.55–0.62	0.42–0.48
				

**Table 4:** Mean scores (±SD) and range of scores of the total THI scale and subscales of the Persian translation (THI-P) and the original version (THI-US).

	THI-total	Functional	Emotional	Catastrophic
**THI-P (mean) (n=102)**	40.4 ± 26.5	22.0 ± 12.9	11.7 ± 9.2	6.7 ± 5.5
**THI-US (mean) (n=66)**	24.4 ± 20.5	11.0 ± 9.7	8.2 ± 8.4	6.1 ± 4.5
**THI-P (range) (n=102)**	4-96	0-46	0-32	0-20
**THI-US (range) (n=66)**	0-92	0-44	0-32	0-18

**Table 5:** Pearson product-moment correlations among THI-total and subscales of the Persian (THI-P) and the original (THI-US) version

	THI-total	Functional	Emotional	Catastrophic
**THI-total**	1.00 (1.00)	-	-	-
**Functional P (US)**	0.97 (0.92)	1.00 (1.00)	-	-
**Emotional P (US)**	0.97 (0.93)	0.90 (0.75)	1.00 (1.00)	-
**Catastrophic P (US)**	0.93 (0.89)	0.84 (0.65)	0.89 (0.78)	1.00 (1.00)

*Confirmatory Factor Analysis*

A confirmatory factor analysis was conducted to test whether the data could confirm the latent variables represented by the functional, emotional, and catastrophic subscales of the original version of the THI. The data were analyzed using a principal components factor analysis with varimax rotation. Because the KMO sampling adequacy was 0.930, the items were considered acceptable for factor analysis. The eigenvalues for these factors were 12.88 (Factor 1), 2.04 (Factor 2), and 1.08 (Factor 3). Factor 1 explained 51.52% of the variance, Factor 2 explained 8.16% of the variance, and Factor 3 explained 4.34% of the variance. To ensure distinct subscales, the differences between the highest and second-highest factor loadings had to exceed 0.20 to be retained in the analysis. In the three-factor solution, 10 items were loaded on more than one factor, with the remaining three factors consisting of 5, 5 and 5 items, respectively. The results are given in Table 6. 

**Table 6:** Results of the three-factor solution of the 25 items of the Persian translation (THI-P) of the Tinnitus Handicap Inventory (THI)^*^

Scale	item		Factor		
			1	2	3
**F**	24	Does your tinnitus get worse when you are under stress?	0.90		
**E**	21	Because of your tinnitus, do you feel depressed?	0.84		
**E**	25	Does your tinnitus make you feel insecure?	0.82		
**E**	22	Does your tinnitus make you feel anxious?	0.80		
**C**	23	Do you feel that you can no longer cope with your tinnitus?	0.75		
**C**	5	Because of your tinnitus, do you feel desperate?	0.63		
**E**	10	Because of your tinnitus, do you feel frustrated?	0.60		
**F**	18	Do you feel it difficult to focus your attention away from your tinnitus?	0.55		
**C**	11	Because of your tinnitus, do you feel that you have a terrible disease?	0.54		
**C**	19	Do you feel that you have no control over your tinnitus?	0.52		
**E**	6	Do you complain a great deal about your tinnitus?	0.50		
**F**	4	Does your tinnitus make you feel confused?	0.39		
**F**	15	Because of your tinnitus, is it difficult for you to read?		0.76	
**F**	14	Because of your tinnitus, do you find that you are often irritable?		0.72	
**F**	7	Because of your tinnitus, do you have trouble falling asleep at night?		0.71	
**E**	16	Does your tinnitus make you upset?		0.64	
**E**	3	Does your tinnitus make you angry?		0.61	
**F**	20	Because of your tinnitus, do you feel tired?		0.56	
**F**	2	Does the loudness of your tinnitus make it difficult to hear people?			0.77
**F**	9	Does your tinnitus interfere with your ability to enjoy social activities?			0.71
**F**	1	Because of your tinnitus, is it difficult for you to concentrate?			0.65
**F**	12	Does your tinnitus make it difficult for you to enjoy life?			0.65
**F**	13	Does your tinnitus interfere with your job or household responsibilities?			0.62
**E**	17	Do you feel that your tinnitus has placed stress on your relationship?			0.57
**C**	8	Do you feel as though you cannot escape your tinnitus?			0.57

*Split Sample Validity*

We validated our analysis by conducting an analysis on each half of the sample. We compared the results of these two split-sample analyses with the analysis of the full data set. All of the communalities in both validation samples met the criteria. The pattern of loadings for both validation samples was the same, and the same as the pattern for the analysis using the full sample. This validation analysis supports a finding that the results of this principal component analysis were generalizable to the population represented by this dataset.

*Construct Validity*

Table 2 summarizes the correlations among scores on the THI-P for tinnitus severity and psychological distress. The mean VAS for tinnitus severity was 6.33 (SD=2.04). In 62 cases (59.8%) VAS was more than 5. A significant correlation was observed between THI-P and tinnitus severity according to the VAS (ρ=0.479, P= 0.001).

The score for the BDI ranged from 0 to 39 (mean=4.7). In 19 cases (18.6%), depressive symptoms were reported. The STAI measures both anxiety as a general trait and the here-and-now level of perceived anxiety. The mean scores for two questionnaires were 41.1 and 44.5, respectively. In 41 (40.2%) and 56 cases (54.9%), state and trait anxiety, respectively, were pathologic. We found a high correlation between the BDI and STAI scores and tinnitus handicap scores of participants with tinnitus (ρ = 0.66 and ρ = 0.69–0.77, respectively).

## Discussion

The results of the present investigation demonstrate that the Persian translation of the THI (THI-P) has a good internal consistency and reliability for the total scale; as also demonstrated in a previous study (23). THI-P had the same internal reliability as the original version (α = 0.93). The internal reliability of the functional (α= 0.91) and emotional (α=0.91) subscales of the THI-P were similar to those of the original THI (α=0.86 and α=0.87, respectively). The internal reliability of the catastrophic subscale of THI-P (α=0.80) was higher than that reported for the original THI version (α= 0.68). 

A well-known measurement parameter for internal consistency is Cronbach’s α coefficient, which means that all test items measure the same construct. As a rule of thumb, values greater than 0.7 show good internal consistencies and values greater than 0.9 show very high internal consistencies (30). To determine whether each item was consistent with the average direction of the other items, an item–total correlation test was performed. If the correlation (calculated using Pearson’s correlation coefficient) between one single item and the total score without this item has a low correlation (<0.3) (31), then the item does not measure the same construct as the others. For the THI-P questionnaire, item 2 showed lower item–total correlations (0.42) than other items; which is in accordance with previous findings reported by Newman et al. (7). These data indicate that hearing difficulties due to tinnitus loudness (item 2) were only moderately correlated with tinnitus handicap. 

THI-P had good external reliability for the total score and the subscale scores (total Pearson correlation, 0.98; functional, emotional, and catastrophic Pearson correlations were 0.98, 0.98, and 0.94, respectively). Items 4 and 23 demonstrated the lowest degree of test–retest reliability.

The high inter-correlations found between the subscales of the THI were similar to those found for the original version. This finding calls into question whether the subscales represent three distinct, underlying latent variables, and it should be mentioned that the three subscales of the THI were not created by a factor analysis (7).

Two points concerning the construct validity of a questionnaire are important to note. First, although researchers often describe their instruments as “validated,” construct validity is an estimate of the extent to which variance in the measure reflects variance in the underlying construct. Second, the extent of observed associations with measures of other match theoretical predictions shows how it is associated with those variables (32). To assess the construct validity, the original THI developers used the BDI, Modified Somatic Perception Questionnaire, and symptom rating scales. Weak correlations were observed between THI and both the BDI and Modified Somatic Perception Questionnaire. Significant correlations were observed between the THI and symptom rating scales. The creators of the Portuguese, Italian, and Cantonese versions of the THI evaluated validity by testing correlations between THI translations and other questionnaires. The Portuguese THI was correlated to the BDI with a Pearson correlation coefficient of 0.68, thus confirming its validity (16). The Italian version correlated well with the MOS 36-Item Short Form Health Survey (SF-36) and the Hospital Anxiety and Depression Scale (HADS), providing evidence of good construct validity (17, 33, 34). THI-CH scores significantly correlated with the anxiety and depression scores of the HADS (20). The results of our confirmatory factor analysis seem to reinforce this doubt, because 10 out of the 25 items were loaded on more than one factor, and the items loading on the remaining three factors only partially represented the subscales suggested by Newman et al (7). Factor analysis of the THI-P yielded similar results to the factor analyses performed on other versions of the THI. Factor analysis of THI-DK (13), the Italian version of the THI (17), THI-CM (19), and THI-CH (20) also revealed unifactorial structures. In the validation study for THI-DK, researchers stated that the three factors only partially matched the items in the original three subscales (13). Factor analysis of the Italian version of the THI indicated that the first, second, and third factors accounted for 35.9%, 7.8%, and 7.5% of the variance, respectively (17). Factor analysis of THI-CM demonstrated that the first, second, and third factors accounted for 38.9%, 6.5%, and 5.0% of the variance, respectively (19). Factor analysis of THI-CH demonstrated that the first, second, and third factors accounted for 41.9%, 6.87%, and 5.87% of the variance, respectively (20). Factor analysis of the original THI version performed in 2003 indicated that three factors could explain 52.8% of the variance, and adding more factors contributed little to the explanation of variance. Additionally, the majority of items were loaded on the first factor (35). The unifactorial structure of the original version was demonstrated.

As was observed for the original version, the Persian version of THI does not appear to be affected by age, gender, or hearing loss. These findings were similar to those found by Mahmoudian et al (23).

Our results showed moderate-to-strong relationships between the THI and depression as measured by the BDI. These findings are in agreement with earlier reports of a relationship between depression and tinnitus severity (36, 37). In contrast, only a weak relationship between THI and depression was observed for the original version. This difference between the Persian and the original sample may be explained by the higher mean THI-total scores (40.4 ± 26.5) of the Persian sample compared with the original US sample (THI-total: 24.4 ± 20.5). Strong-to-moderate correlations were also observed between the THI-P and the STAI questionnaires. Similar correlations were generally observed for THI-total scores and scores on the three subscales. This finding suggests that the subscales may not necessarily measure three distinct factors. The high correlation between the three subscales in the confirmatory factor analysis is in favor of the above finding. These findings are in concordance with the earlier Zachariae study which failed to validate the three distinct factors (13). 

We showed a clear linear relationship between a VAS estimate of tinnitus severity and the corresponding scores for the THI-P questionnaire. This demonstrates that total score could distinguish between different grades of tinnitus severity.

The most important specifications of an instrument for assessment include repeatability, consistency, and validity of the obtained scores. We evaluated reliability of the THI-P from two aspects of the test–retest reliability and Interclass Correlation Coefficient (ICC). The other aspect of consistency was related to the correlation between test–retest which was evaluated by the Pearson correlation coefficient. We obtained a value of 0.984 in the Persian THI version, consistent with significant correlation in retest. Comparison of consistency in the finding of this research with the original English version using Cronbach’s α coefficient revealed a significant correlation of the Persian version (0.96) with the English version (0.93) (7). The present study proved the internal consistency/coherency of the THI-P. This demonstrates that the THI-P is satisfactory for application in clinical/research environments. 

With respect to the subscales of THI-P, our data suggest that the items of the subscales included in the original version of the THI may not represent distinct underlying latent variables, and further studies using the THI and other similar measures are needed to clarify whether specific tinnitus distress measures can be identified. Since the design of this study did not allow for the testing of different subset of patients with tinnitus, it is recommended that the relationship between the THI-P and psychopathological problems be investigated.

## Conclusion

In conclusion, the results of the study suggest that the THI-P is a reliable measure of tinnitus-related handicaps. The reliability of THI-P is in line with that of the original English version. Consistent with previous findings reported by Mahmoudian (23), the THI-P has high internal consistency, adequate test–retest reliability and adequate reproducibility. In addition, we found high construct validity, and concurrent validity. Thus, we conclude that the THI-P is a reliable and valid tool to assess tinnitus-related handicap in Persian-speaking adults. However, it appears that the subscale items of the THI do not represent distinct underlying latent variables, and further studies using the THI and other similar measures are needed to clarify whether specific tinnitus distress measures can be identified.
